# Schisandra Fruit Vinegar Lowers Lipid Profile in High-Fat Diet Rats

**DOI:** 10.1155/2020/7083415

**Published:** 2020-09-03

**Authors:** Rui Yuan, Guangren Sun, Jiaqi Gao, Zepeng Yu, Chunyan Yu, Chunmei Wang, Jinghui Sun, He Li, Jianguang Chen

**Affiliations:** ^1^Department of Pharmacology, College of Pharmacy, Beihua University, Jilin 132013, China; ^2^Department of Food Science, College of Forestry, Beihua University, Jilin 132013, China; ^3^Department of Pathology, College of Medicine, Beihua University, Jilin 132013, China

## Abstract

Hyperlipidemia and its associated obesity, hepatic steatosis, and NAFLD are worldwide problems. However, there is no ideal pharmacological treatment for these. Therefore, the complementary therapies that are both natural and safe have been focused. Healthy foods, such as fruit vinegar, may be one of the best choices. In this study, we made a special medicinal fruit vinegar, Schisandra fruit vinegar (SV), and examined its lipid-lowering effects and the underlying mechanisms in a high-fat diet rat model. The results showed that SV significantly reduced the body weight, liver weight, liver index, the serum triglyceride (TG), total cholesterol (TC), low-density lipoprotein cholesterol (LDL-C), free fatty acid (FFA), aspartate aminotransferase (AST), alanine aminotransferase (ALT), and malondialdehyde (MDA), increased the content of high-density lipoprotein cholesterol (HDL-C) and the activity of superoxide dismutase (SOD), upregulated the expressions of peroxisome proliferative activated receptor (PPAR-*α*), peroxisomal acyl-coenzyme A oxidase 1 (ACOX1), and carnitine palmitoyltransferase 1 (CPT1) proteins, increased the contents of key component of antioxidant defense NF-E2-related factor 2 (NRF2) and its downstream heme oxygenase-1 (HO-1) protein, and downregulated the expression of Kelch-like ECH-associated protein 1 (KEAP1). These results suggest that SV has weight loss and lipid-lowering effects in HFD rats, which may be related to its upregulation of the expressions of *β*-oxidation -elated PPAR-*α*, CPT1, and ACOX1 and the regulation of the expressions of antioxidant pathway-related KEAP1-NRF2-HO-1. Therefore, all these data provide an experimental basis for the development of SV as a functional beverage which is safe, effective, convenient, and inexpensive.

## 1. Introduction

Excessive consumption of energy-dense foods is very popular in the western countries as well as in some developing countries. As consequences, obesity, hyperlipidemia, hepatic steatosis, and nonalcoholic fatty liver disease (NAFLD), even cardiovascular diseases, have also become popular. However, to date, there is no ideal pharmacological treatment for hyperlipidemia and its associated consequences [1]. Because there are lots of possible adverse effects of conventional medical therapies, there has been a growing interest in the complementary therapies which were both natural and safe [2]. Thus, in the field of food science, finding food or beverage with the function to prevent hyperlipidemia and obesity induced by excessive consumption of energy-dense has been important nutritionally and has been focused. Vinegar may be one of the best choices. Vinegar is a sour-tasting condiment throughout the world and very popular in China, South Korea, and Japan. According to the raw materials used, vinegar can mainly be classified as grain vinegar and fruit vinegar, both are obtained from the anaerobic conversion of sugars to ethanol by yeasts first and then aerobic oxidation of ethanol to acetic acid by specific bacteria. Vinegar can not only improve taste and digestion of food but can also prevent and treat obesity, hyperlipidemia, infection, cold, stomach illness, hypertension, liver disease, diabetes, cancer, and aging, etc. [3]. *Schisandra* vinegar (SV), we made and used in the present study, is different from the grain vinegar and commonly fruit vinegar, because its raw material is from the fruits of *Schisandra*, which is a well-known traditional herbal medicine and has been cultivated and therapeutically used for thousands of years in China, Korea, Japan, and Russia [4]. *Schisandra* has been proved to have lipid-lowering effect [5], lower blood sugar, and enhance the body's antioxidant capacity. Our previous studies also found that its lignans, polysaccharides, and other active ingredients also have lipid-lowering effects [6, 7]. Therefore, we may classify SV as a medicinal fruit vinegar. By taking advantage of *Schisandra* as both food and medicine, we specially made SV and examined its lipid-lowering and weight loss effects in high-fat diet (HFD) rats through weighting and relative biochemical indicators. In addition, in order to further explore the underlying mechanism, we examined the expressions of *β*-oxidation related PPAR-*α*, CPT1, and ACOX1 and antioxidant pathway related KEAP1-NRF2-HO-1. We hope this study will provide a scientific evidence for SV to be developed as a potential beverage to promote the recovery or prevent hyperlipidemia.

## 2. Materials and Methods

### 2.1. Experimental Animals

Forty male SPF Wistar rats (210 g–250 g) were purchased from Changchun Yisi Laboratory Animal Technology Co., Ltd. (Changchun, China; Certificate number: SCXK (Ji) 2016-0003). The rats were fed for 6 weeks in separate cages with a good ventilation, at a constant temperature (21–23°C), a constant humidity (45%–65%), and with light and dark cycles for 12 h each. Animal experiments were approved by the Institutional Animal Care and Use Committee (IACUC) of Beihua University with the permit number of CPBHU IACUC2019-006, and all experimental procedures were carried out in accordance with Guidelines on Ethics and Use of Laboratory Animal of Beihua University.

### 2.2. Instruments

Full wavelength ELISA (EPOCH, BIOTEX, USA); electronic balance (LD310-2, Shenyang, China); high performance liquid chromatography (LC-2010A, SHIMADZU, Japan); chemiluminescence gel imaging system (Surwit Technology Inc., Beijing, China); automatic biochemical analyser (Tecan Group, Switzerland); laboratory pH meter (Metler-Toledo Instrument Co., Ltd., China); low-speed centrifuge (Xinsheng Biotechnology Co., Ltd., Ningbo, China); optical microscope (OLYMPUS, Japan); Ultraviolet-Visible Spectrophotometer (Beijing Purkinje General Instrument Co., Ltd, Beijing, China).

### 2.3. Chemicals and Reagents

*Schisandra chinensis* was obtained from Jian Schisandra Seedlings Base of Jilin Province. SV (Schisandra Development and Industrialization Engineering Research Center of Jilin Province, Jilin, China); TG, TC, FFA, HDL-C, LDL-C, AST, ALT, MDA, and SOD detection kits (Nanjing Jiancheng Bioengineering Institute, Nanjing, China); rabbit anti-rat PPAR-*α*, ACOX1, CPT1, KEAP1, NRF2, and HO-1 antibodies (Abetclonal Company, San Francisco, USA); cholesterol, sodium cholate, calcium hydrogen phosphate, and casein (Anhui Tianqi Chemical Technology Co., Ltd., Anhui, China); egg yolk powder (Nanjing Xiaolongshan Laboratory Animal Reproduction Center, Nanjing, China); HFD containing 20% sucrose, 15% lard, 10% casein, 2% cholesterol, 0.6% calcium hydrogen phosphate, and 0.2% sodium cholate; and normal diet (Changchun Yisi Laboratory Animal Co., Ltd., Changchun, China).

### 2.4. Preparation of SV

The dry mature fruit of schisandra was rinsed with water and drained. Then, the clean and dried schisandra fruit was boiled in a steamer for 1 h. Every 1 kilogram of the boiled fruit was added with 1 mL pectinase and was mixed with distilled water at a ratio of 1 : 10 at 45°C for 1.5 h to produce a schisandra jam. Firstly, the jam was converted into schisandra fruit wine by adding 7% yeast activating liquid at 28°C for 3 days. Then, the schisandra fruit wine was adjusted to an alcoholicity of 8% (V/V) and inoculated with 6% acetic acid bacteria solution with the fermentation ventilation of 0.15 L·min^−1^ at 30°C for 15 days. Finally, the fruit wine was converted into SV by acetic acid fermentation. This fruit vinegar was filtered and sterilized for the experimental use.

### 2.5. Analysis of Acidity and Components of SV

The acidity of SV was measured by a pH meter. The content of acetic acid was determined by acid-base neutralization and titration according to the Pharmacopoeia of the People's Republic of China. The content of polysaccharide was measured by phenol-sulfuric acid method. The content of schisandrin A was determined by high performance liquid chromatography (HPLC) according to “Determination of schisandrin A in schisandra health food” in the Pharmacopoeia of the People's Republic of China [8].

### 2.6. Experimental Protocol

**Figure 1:**
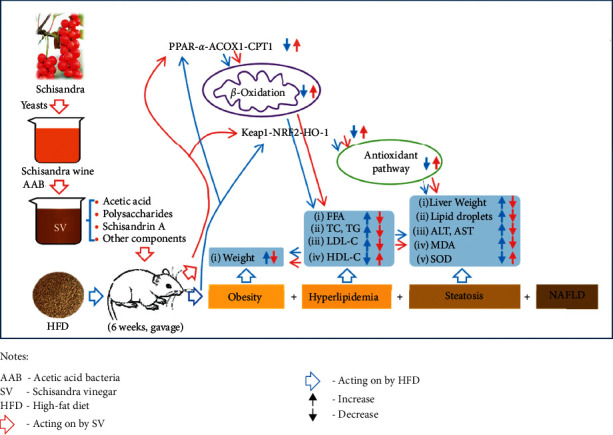
Experimental protocol. Schisandra vinegar (SV) was administered by gavage to male SPF Wistar rats on high-fat diet for 6 weeks. Then after, liver weights, liver tissue histological changes, serum biochemical indicators, and antioxidation relative proteins, including PPAR-*α*, ACOX1, CPT1, KEAP1, NRF2, and HO-1, were measured.

As shown in Figure 1, forty male SPF Wistar rats were randomly and evenly divided into five groups, i.e., blank control group (CON, normal diet, distilled water gavage), model group (MOD, HFD, distilled water gavage), low-dose SV group (SVL, HFD, 25% SV gavage), medium-dose SV group (SVM, HFD, 50% SV gavage), and high-dose SV group (SVH, HFD, 100% SV gavage), as shown in Table 1. All rats were undergoing corresponding administrations for six weeks and weighed once a week. Then, the rats were fasted for 12 h but fed water. Finally, all rats were anesthetized with ether and their abdominal aorta blood and liver samples were collected for the detection or analysis of liver weights, liver tissue histological changes, serum biochemical indicators, and antioxidation relative proteins, including PPAR-*α*, ACOX1, CPT1, KEAP1, NRF2, and HO-1.

### 2.7. Calculation of Liver Index

The liver index (%) was calculated as wet liver weight (g)/body weight (g) × 100%.

### 2.8. Detection of the Serum Lipid Profile and Biochemical Indicators

The collected blood was centrifuged at 4000 r/min for 10 min to obtain the serum. The serum TG, TC, FFA, HDL-C, LDL-C, AST, ALT, and MDA contents and SOD activity were detected according to the instructions of corresponding kits.

### 2.9. Liver Tissue Histological Analysis

Histology of liver tissues was performed routinely. The liver was rinsed with PBS and fixed in 10% buffered formalin for 24 h. Then, the liver was embedded in paraffin, sectioned into 7 ± 2 *μ*m, deparaffinized, and rehydrated using the standard techniques, then stained with hematoxylin and eosin. The morphology of the liver was observed using microscope (New York, USA) at 200 times magnification.

### 2.10. Western Blot Detection

The liver tissue was homogenated with lysis buffer at a ratio of 1 : 9, cracked on ice for 1 h, and centrifuged for 10 min. The total protein concentration in the supernatant was determined according to the instructions of BCA kit. The absorbance was measured at 562 nm and the standard curve was drawn for the calculation of the protein concentration. SDS-PAGE gel electrophoresis of 10% separating gel and 5% concentrating gel was used to isolate the proteins. After electrophoresis, the proteins were transferred onto PVDF membranes for 2 h. The blocking buffer (TBS-T buffer containing 5% skimmed milk powder) was used for blocking the membrane for 1 h, and then the blocking buffer was discarded. PPAR-*α*, ACOX1, CPT1, KEAP1, NRF2, and HO-1 antibodies were prepared with TBS-T in certain proportions. The antibodies and the above PVDF membranes were placed in the same container, which were placed in a chromatographic chamber at 4°C for 12 h. The membranes were incubated with the second antibodies in a shaker at room temperature for 2 h. Then, the membranes were washed and developed with ECL developer by a gel imaging system.

### 2.11. Statistical Analysis

The data were expressed as mean ± standard deviation (mean ± SD). SPSS 20.0 statistical software was used for the statistical analysis of One Way ANOVA. *T*-test was used to compare the two groups. *P* < 0.05was considered to be statistically significant.

## 3. Results

### 3.1. The Main Chemical Properties of SV

The main ingredient of the grain or fruit vinegar is acetic acid. However, in SV, a medicinal fruit vinegar, besides acetic acid, there were other active components that originated in Schisandra fruit. The measurement results showed that SV contained acetic acid of 34.83 ± 1.29 mg/ml, total polysaccharides of 1.49 ± 0.09 mg/ml, and schisandrin A of 0.15 mg/ml. In addition, pH of SV was 3.25 ± 0.01, within the range of common vinegar.

### 3.2. SV Decreased the Body Weight and Liver Weight

As shown in Table 2, a gradual increase of body weight and liver weight is a mark for the HFD animal model, which also represents the progressive progress of NAFLD. In the present study, HFD increased both body weight, liver weight, and liver index significantly in MOD group compared with CON group (*P* < 0.05;*P* < 0.01). However, SV decreased the liver weight significantly at medium-dose but decreased body weight, liver weight, and liver index significantly at high dose. These results suggested that SV have a slight but significant effect on weight loss and promote the recovery of liver injury in steatosis.

### 3.3. SV Lowered Lipid Profile

**Figure 2:**
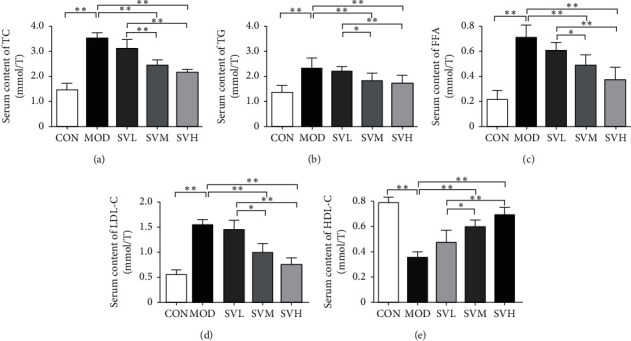
The effect of SV on the serum lipid profile in HFD rats. After the gavage of SV to HFD rats for 6 weeks, the serum TG (a), TC (b), LDL-C (c), FFA (d), and HDL-C (e) levels were measured. The data are shown as the mean ± SD, *n* = 8. Compared between different groups, ^*∗*^*P* < 0.05,^*∗∗*^*P* < 0.01.

As shown in Figure 2, excessive consumption of energy-dense foods such as a HFD is the primary cause of obesity and hyperlipidemia. HFD in the present study also induced high level in lipid profile. HFD in MOD group increased the serum TG, TC, LDL-C, and FFA levels (A, B, C, and D, respectively), but decreased serum HDL-C level (E) significantly in comparison to CON group (*P* < 0.01). However, SV at medium or high dose decreased the serum TG, TC, LDL-C, and FFA levels (A, B, C, and D, respectively), but increased serum HDL-C level (E) significantly in comparison to the MOD group (*P* < 0.01). In addition, there were significant differences among the three SV groups in decreasing serum TC, TG, FFA, and LDL-C levels and increasing HDL-C level (*P* < 0.05 or *P* < 0.01), suggesting that SV lowers the lipid profile dose-dependently.

### 3.4. SV Decreased the Serum Transaminases

**Figure 3:**
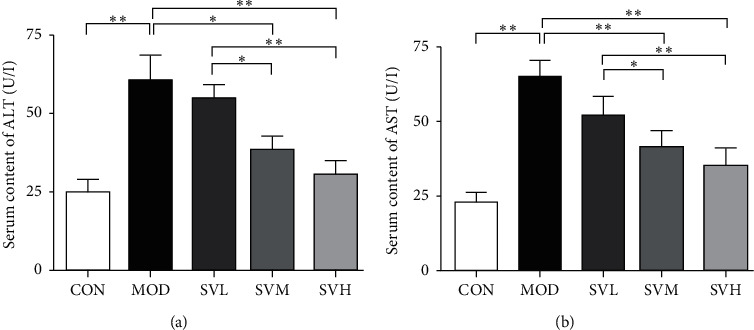
The effect of SV on the serum ALT and AST contents in HFD rats. After the gavage of SV to HFD rats for 6 weeks, the serum ALT (a) and AST (b) contents were measured. The data are shown as the mean ± SD, *n* = 8. Compared between different groups, ^*∗*^*P* < 0.05, ^*∗∗*^*P* < 0.01.

As shown in Figure 3, ALT and AST are mainly distributed in liver cells. If the liver is damaged, these transaminases will leak into the bloodstream from liver cells, therefore, the elevated levels of ALT and AST are the signs of liver injury. In the present rat model, 6 weeks of HFD produced the fat deposition in the liver and damaged it, resulting in a significant increase of ALT and AST levels in MOD group (*P* < 0.05,*P* < 0.01), whereas SV at medium and high dose decreased the serum ALT and AST significantly in comparison to MOD group (*P* < 0.05,*P* < 0.01). Moreover, SV also showed its effect in a dose-dependent manner.

### 3.5. SV Increased the Serum SOD Activity and Decreased the Serum MDA Content

**Figure 4:**
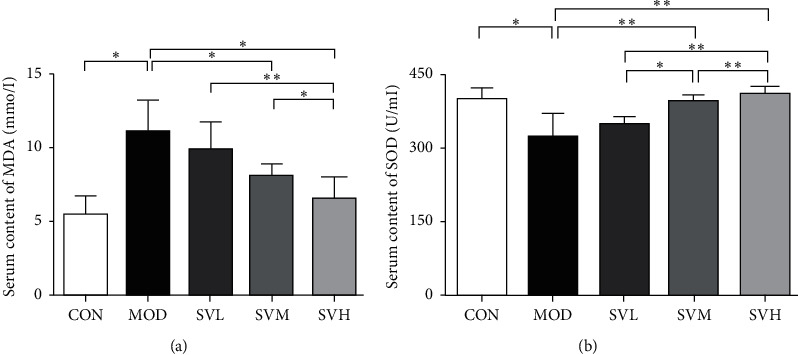
The effect of SV on the serum MDA content and SOD activity in HFD rats. After the gavage of SV to HFD rats for 6 weeks, serum MDA content (a) and SOD activity (b) were measured. The data are shown as the mean ± SD, *n* = 8. Compared between different groups, ^*∗*^*P* < 0.05, ^*∗∗*^*P* < 0.01.

As shown in Figure 4, the antioxidant activity in this study was evaluated by the measurement of serum SOD activity and MDA content. SOD acts as the first line of defense oxidative stress that converts oxygen radicals (O^2−^) to hydrogen peroxide (H_2_O_2_) and dioxygen. MDA is produced by the product of unsaturated lipid peroxidation and considered as toxic molecule and biological marker of oxidative stress [9]. Our results showed that HFD decreased SOD activity and increased MDA content in the MOD group significantly when compared with CON group. In contrast, SV at medium and high dose increased SOD activity and decreased MDA content significantly in comparison to the MOD group. Overall, our data suggest that SV treatment can regulate cellular antioxidant elements to ameliorate HFD induced oxidative stress significantly and dose-dependently.

### 3.6. Histological Changes in the Rat Livers

**Figure 5:**
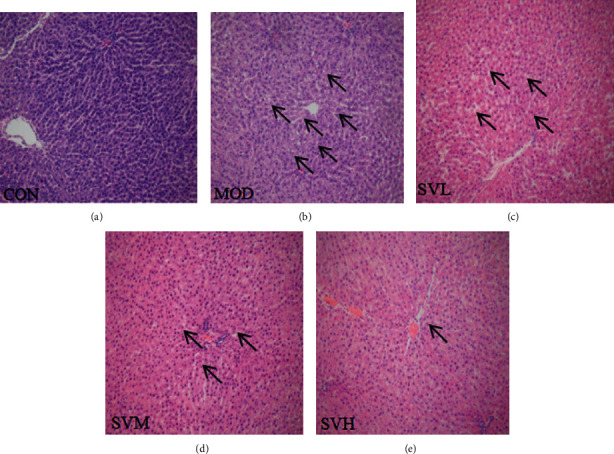
The effect of SV on the liver tissue histological alterations in HFD rats. After the gavage of SV to HFD rats for 6 weeks, the liver tissue histological alterations in HFD rats were observed using microscope. The arrows indicate the fat vacuoles. Hematoxylin and eosin, 200X. (a) CON. (b) MOD. (c) SVL. (d) SVM. (e) SVH.

As shown in Figure 5, pathological changes can directly reflect the accumulation of lipid droplets and inflammatory infiltration in the liver [10, 11]. The hepatic lobular structure of the CON group is intact, the central vein is located in the center of the hepatic lobule, the surrounding hepatocytes were radially arranged, and the structure of the hepatic sinus was clear. The hepatocyte was polygonal, the nucleus was located in the center and blue stained, the chromatin was clear, and the cytoplasm was homogenized. However, in the MOD group, the lobular structure was intact, but the hepatocytes were swollen and fat vacuoles of varying sizes appeared in the cytoplasm, as indicated by the arrows. The nucleus was dislocated, the chromatin was clear, and the hepatic sinus was compressed. Inflammatory cell infiltration could be seen in the portal area. In the SV low and middle dose groups, the hepatic lobular structure was intact, and fat vacuoles, the number of hepatocytes with steatosis, and the hepatic sinus compression were apparently reduced. The nuclei were deep stained, and the hepatic sinus compression and the inflammatory cells in the hepatic sinus area were also clearly reduced. In the SV high-dose group, the hepatic lobular structure was intact and the fat vacuoles of hepatocytes and the inflammatory cells were obviously reduced. The nucleus chromatin was clear, and the hepatic sinus compression was also alleviated. From the pathology, we can see that the improvement effect of the high-dose SV on the pathological changes of the livers is clearly better than those of the low and medium-dose SV. Therefore, SV exerts its beneficial effects on both reducing blood lipids and improving liver tissue.

### 3.7. SV Upregulated the Expressions of PPAR-*α*, CPT1, and ACOX1 Proteins

**Figure 6:**
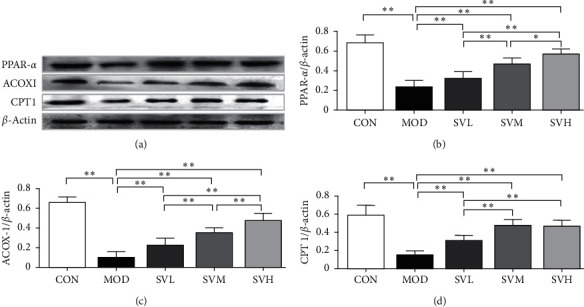
Effects of SV on the expressions of PPAR-*α*, ACOX1, and CPT1 proteins of the livers in HFD rats. After the gavage of SV to HFD rats for 6 weeks, the expressions of PPAR-*α*, ACOX1, and CPT1 proteins were measured. (a) Electrophoretogram, (b) PPAR-*α*, (c) ACOX1, (d) CPT1. The data are shown as the mean ± SD, *n* = 8. Compared between different groups, ^*∗*^*P* < 0.05, ^*∗∗*^*P* < 0.01.

As shown in Figure 6, PPAR-*α* is a nutritional sensor, which allows adaptation of the rates of FFA catabolism, lipogenesis, and ketone body synthesis in response to feeding and starvation. PPAR-*α* and its target genes, ACOX1 and CPT1, are involved in fatty acid *β*-oxidation in the liver [12, 13]. Their elevation can activate the process of *β*-oxidation, resulting in the FFA catabolism and lipolysis. HFD decreased the hepatic expression levels of PPAR-*α*, ACOX1, and CPT1 in the MOD group significantly in comparison to the CON group (*P* < 0.01); however, SV at all three doses increased the hepatic expression levels of all of them in a dose-dependent manner and significantly when compared with the MOD group (*P* < 0.01). These results suggest that SV lowers lipid profile partly through PPAR-*α* and its target genes ACOX1 and CPT1 in activating *β*-oxidation in the liver.

### 3.8. SV Regulated the Expressions of KEAP1, NRF2, and HO-1 Proteins

**Figure 7:**
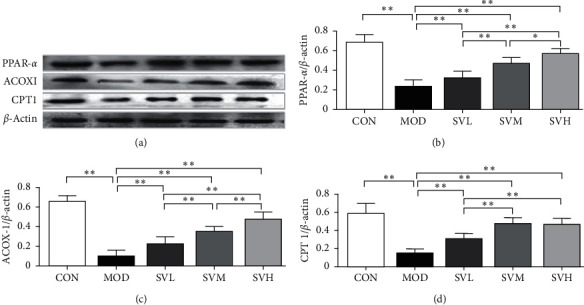
Effects of SV on the expressions of KEAP1, NRF2, and HO-1 proteins of the livers in HFD rats. After the gavage of SV to HFD rats for 6 weeks, the expressions of KEAP1, NRF2, and HO-1 proteins were measured. (a) Electrophoretogram, (b) KEAP1, (c) NRF2, (d) HO-1. The data are shown as the mean ± SD, *n* = 8. Compared between different groups, ^*∗*^*P* < 0.05, ^*∗∗*^*P* < 0.01.

As shown in Figure 7, KEAP1-NRF2-HO-1 is the main antioxidant pathways [14]. When reactive oxygen species generation and lipid peroxidation occur in mitochondria, this pathway is activated and modulate genes expression of cytoprotective proteins and enzymes, which decreases reactive oxygen species levels and tissue injury. We examined the effect of SV on this pathway. Our results showed that, in the MOD group, the expression of KEAP1 protein was significantly higher (*P* < 0.01) and the expressions of NRF2 and HO-1 proteins were significantly lower (*P* < 0.01) than those of the CON group. On the contrary, SV at all doses decreased the expression of KEAP1 protein and increased the expressions of NRF2 and HO-1 proteins significantly compared with the MOD group (*P* < 0.05 or *P* < 0.01). These results suggest that SV not only exerts the direct lipid-lowering effect, but also protects the consequent harmful effect induced by reactive oxygen species generation and lipid peroxidation through activating KEAP1-NRF2-HO-1 antioxidant pathway.

## 4. Discussion

In this study, SV treatment successfully prevented the HFD induced increases in the body weight, liver weight, and lipid profile and improved histological alterations in rat model. Furthermore, SV also regulated fatty acid *β*-oxidation related PPAR-*α*, CPT1, and ACOX1 and antioxidant pathway related KEAP1-NRF2-HO-1 protein expressions.

In our study, HFD was used to produce the rat model with significant elevated lipid profile, increase in body weight, histological changes, and some changes in important factors related to fatty acid *β*-oxidation and antioxidant pathway. All these made this model a valuable research tool to evaluate the potential effect of medicine or complementary treatment. It may be “the optimal window” for the conventional and/or complementary interventions in the early stage, such as slight weight gain, elevated lipid profile, and hepatic steatosis, rather than in the more severe last stage, such as nonalcoholic steatohepatitis (NASH), NAFLD, or even cirrhosis and hepatocellular carcinoma. In fact, there are currently few pharmacological options available to treat NAFLD. Therefore, complementary therapies, such as lifestyle modification, herbs, and their bioactive compounds, etc., are becoming more and more attractive in the prevention and treatment of the manifestations in the early stage of NAFLD. Healthy food may be one of the best choices. Accordingly, in the present study, we made a special medicinal fruit vinegar and investigated its lipid-lowering effects and the mechanisms underlying.

Vinegar has been widely used as a dietary spice and natural remedy for various ailments in folk medicine all over the world now. The earliest known use of vinegar dates to more than 10000 years ago [13]. Flavored vinegar has been produced and sold as a commercial product for approximately 5000 years. The father of medicine Hippocrates (420 BC) once used vinegar for wound care [14]. Vinegar may be classified by their raw materials as grain vinegar, mainly from rice and wheat, or as fruit vinegar, mainly from grape, apple, and coconut [14, 15]. Both grain vinegar and fruit vinegar possess a variety of physiological functions, such as antibacteria, anti-infection, antioxidation, blood glucose control, lipid metabolism regulation, weight loss, and anticancer activities [16]. The active compounds identified in vinegar mainly include organic acids, polyphenols, melanoidins, ligustrazine, caffeoyl sophorose, and tryptophol [15]. Among them, researches reported that acetic acid played key roles in the regulation of lipid metabolism and weight loss due to inhibiting the fatty acid and lipid synthesis, increasing the oxygenolysis and secretion of lipids, increasing postprandial satiety, and increasing energy consumption [17, 18]. However, in SV, we used schisandra fruits instead of common fruits; besides acetic acid, there are some other active components, such as total polysaccharides and schisandrin A, which make SV unique among other common fruit vinegars. Our study and other previous studies have shown that total polysaccharides and schisandrin A had significant lipid-lowering effects [5, 6]. Therefore, within SV, lignans (include schisandrin A), polysaccharides, and acetic acid, together with the other nutrients, may play a synergetic effect in weight loss and lipid-lowering.

Obesity is characterized by the excessive accumulation and storage of fat in the body and is often associated with hyperlipidemia. About obesity treatment adjuvants, orlistat and sibutramine are the only antiobesity drugs approved by the US Food and Drug Administration (FDA). However, these drugs are associated with lots of side effects [19]. Vinegar can be labeled safe and effective by default but needs to be confirmed by scientific evidence. The results of the present study showed that SV could manage body weight and control serum lipid profile in the HFD rats. In our HFD rat model, even the body weights were not increased dramatically, the less and slow weight loss may be beneficial to avoid a worsening of fibrosis and hepatocytes necrosis, and then, successful in the long term [20]. The material basis of these actions of SV might come from its plentiful acetic acid, schisandrin, and total polysaccharide.

In order to further explore the lipid-lowering mechanism of SV in HFD rats, the protein expressions of FFA *β*-oxidation related PPAR-*α*, CPT1, and ACOX1, and antioxidant pathway related KEAP1-NRF2-HO-1 were detected. PPAR-*α*, the key enzyme in the oxidative metabolism of FFA, can regulate the oxidative catabolism of FFA in the liver and maintain the balance of lipid and energy in the liver [1, 21]. The main function of PPAR-*α* is to reduce the TG level, increase the serum HDL-C level, and increase the uptake and oxidative decomposition of FFA. ACOX1 and CPT1, as the downstream genes of PPAR-*α*, are also the rate-limiting enzymes in the oxidation metabolism of fatty acid [22]. The deficit of ACOX1 results in the inability to metabolize very long-chain Lipoyl CoA, the accumulation of long-chain and very long-chain fatty acids in hepatocytes, resulting the accumulation of lipids [23, 24], whereas CPT1, located at the mitochondrial outer membrane through transmembrane area, catalyze the transfer of the acyl group of a long-chain fatty acyl-CoA from coenzyme A to L-carnitine. This process facilitates the beta-oxidation of fatty acids [25]. Therefore, CPT1 can prevent or decrease the fatty acid accumulation in the liver [26, 27]. Our results showed that SV could increase the expression levels of all these three proteins, suggesting that SV may reduce the obesity and hyperlipidemia partly through promoting lipid metabolism.

The liver is the main site of lipid metabolism; high level of fat and cholesterol can destroy the balance of its lipid metabolism, resulting in the deposition of fatty acids and TG in the liver and mitochondrial dysfunction [28]. Because mitochondria are the primary source of cellular ROS, its dysfunction can lead to ROS overproduction. ROS will trigger lipid peroxidation, resulting in more products of peroxidation such as MDA in the liver [3]. Therefore, oxidative stress and lipid metabolic disorders interact to promote the formation of dyslipidemia, obesity, hepatic steatosis, and even NAFLA. KEAP1-NRF2-HO-1 related pathway is considered one of the most important endogenous antioxidant signaling pathways in the body, which can reduce the level of oxidative stress [29, 30]. Under normal physiological conditions, NRF2 maintains low transcriptional activity. However, when the body is stimulated by the electrophilic reagents or ROS, KEAP1 can quickly uncouple with NRF2, which in turn, binds with antioxidant responsive element (ARE) to activate target gene expression and regulate the expression of antioxidant-related proteins, and finally play an antioxidant role [31, 32]. When the KEAP1/NRF2 pathway is activated, the expressions of HO-1 and SOD are upregulated. HO-1 is an important antioxidant enzyme to resist endogenous and exogenous stimuli, can prevent free heme from participating in the oxidation reaction, plays an antioxidant role with bilirubin, the product of its enzymatic hydrolysis, and improves microcirculation [9, 33]. SOD, as the first line of defense oxidative stress, converts oxygen radicals (O^2−^) to hydrogen peroxide (H_2_O_2_) and dioxygen [34]. In this study, HFD of 6 weeks caused downregulation of NRF2 and HO-1 and upregulation of KEAP1 in the model rats. However, SV could downregulate the expression of KEAP1 and upregulate the expressions of NRF2 and its downstream HO-1 as well as SOD. In addition, MDA, a product of lipid peroxidation, was also reduced. As a pair of important indicators to easily evaluate the oxidative stress status, they are often detected in the experimental animals with oxidative stress and hyperlipidemia, showing the decrease of SOD activity and the increase of MDA level [35]. These indicate that SV may enhance the antioxidant capacity by regulating the KEAP1/NRF2/HO-1 pathway.

In conclusion, SV showed its weight loss and lipid-lowering effects in HFD rats. These effects might be a synergistic effect of acetic acid, polysaccharides, schisandrin A, and other active substances contained in SV. The mechanisms underlying might be mainly through two ways: one is upregulation of the expression of FFA *β*-oxidation related PPAR-*α*, CPT1, and ACOX1; the other is the regulation of the expressions of antioxidant pathway related KEAP1-NRF2-HO-1, upregulating KEAP1, and downregulating NRF2 and HO-1. Our research provides an experimental basis for the development of SV as a safe, effective, convenient, and inexpensive functional beverage, especially for the long-term use to treat the slight manifestation in the early stage of NAFLD.

## Figures and Tables

**Table 1:** Rat diets and administration.

Group	Diet	Administration (gavage, 10 ml·kg^−1^)
CON	Normal diet	Distilled water
MOD	High-fat diet	Distilled water
SVL	High-fat diet	25% SV
SVM	High-fat diet	50% SV
SVH	High-fat diet	100% SV

**Table 2:** Effects of Schisandra fruit vinegar (SV) on the body weight, liver weight, and liver index in HFD rats (mean ± SD, *n* = 8).

Group	Body weight (g)	Liver weight (g)	Liver index (%)
CON	313.00 ± 14.45	8.35 ± 0.75	2.77 ± 0.07
MOD	346.00 ± 28.46^##^	10.40 ± 0.42^##^	3.02 ± 0.25^#^
SVL	341.43 ± 42.80	10.18 ± 0.51	3.03 ± 0.51
SVM	323.13 ± 24.01	9.19 ± 0.27^*∗∗*^	2.86 ± 0.22
SVH	318.63 ± 18.94^*∗*^	8.77 ± 0.45^*∗∗*^	2.76 ± 0.21^*∗*^

After SV was intragastrically given for six weeks, the body weight and liver weight of rats in each group were measured, and the liver index was calculated. The values indicate mean ± SD of  8 rats. Differences  between CON  group  and  MOD group  were  determined  using  Student's *t*-test  (^#^*P* < 0.05 and ^##^*P* < 0.01). Differences between MOD group and each SV-treated group  were  assessed  using Student's* t*-test  (^*∗*^*P* < 0.05 and ^*∗∗*^*P* < 0.01).

## Data Availability

The data used to support the findings of this study are included within the article.
